# Programmed death-ligand 1 expression according to epidermal growth factor receptor mutation status in pretreated non-small cell lung cancer

**DOI:** 10.18632/oncotarget.22837

**Published:** 2017-12-01

**Authors:** Akito Hata, Nobuyuki Katakami, Shigeki Nanjo, Chiyuki Okuda, Reiko Kaji, Katsuhiro Masago, Shiro Fujita, Hiroshi Yoshida, Kota Zama, Yukihiro Imai, Yukio Hirata

**Affiliations:** ^1^ Division of Integrated Oncology, Institute of Biomedical Research and Innovation Hospital, Kobe, Japan; ^2^ Department of Contract Research for Clinical Pathology, GeneticLab Co. Ltd., Sapporo, Japan; ^3^ Department of Clinical Pathology, Kobe City Medical Center, General Hospital, Kobe, Japan

**Keywords:** PD-L1, *EGFR* mutation, non-small cell lung cancer, PD-L1 IHC, PD-L1 antibody

## Abstract

**Background:**

Current clinical trials have suggested poorer efficacies of anti-programmed death-1 (PD-1)/PD-ligand 1 (PD-L1) immunotherapies for non-small cell lung cancer (NSCLC) harboring epidermal growth factor receptor (*EGFR*) mutations, implying lower PD-L1 expression in *EGFR*-mutant NSCLC than in *EGFR*-wild type.

**Methods:**

We retrospectively analyzed correlation between PD-L1 expression and *EGFR* status in clinical samples of pretreated NSCLC. PD-L1 immunohistochemistry was performed using the 28-8 anti-PD-L1 antibody for tumor cell membrane staining. H-score was adopted to evaluate both percentage and intensity. We investigated H-scores ≥1, ≥5, and ≥10 as PD-L1+ cut-offs. H-score ≥10 was defined as strong PD-L1+.

**Results:**

We investigated 96 available histologic samples in 77 pretreated patients with NSCLC. Median H-score in *EGFR*-mutant samples (n=65) was 3 (range, 0-150), whereas *EGFR*-wild-type (n=31) was 8 (range, 0-134) (p=0.0075). Using H-scores ≥1, ≥5, and ≥10 cut-offs, incidence of PD-L1+ in *EGFR*-mutant vs. *EGFR*-wild-type samples were: 85% (55/65) vs. 94% (29/31) (p=0.2159); 42% (27/65) vs. 74% (23/31) (p=0.0027); and 22% (14/65) vs. 48% (15/31) (p=0.0074), respectively. Patient-oriented (n=77) univariate analysis for strong PD-L1+ found age of sample (p=0.0226) and *EGFR* mutation status (p=0.0490) as significant factors. Multivariate analysis identified *EGFR* mutation status as the only significant factor (p=0.0121, odds ratio 2.99) for strong PD-L1+. H-scores of PD-L1 expression varied in all 11 cases receiving multiple rebiopsies, and categories of positivity migrated in 10 (91%) of 11 patients.

**Conclusions:**

PD-L1 expression was significantly lower in *EGFR*-mutant NSCLC samples than in *EGFR* wild-type samples. Its expression could be dynamic and affected by age of sample.

## INTRODUCTION

Lung cancer is the leading cause of cancer deaths worldwide. Non-small cell lung cancer (NSCLC) accounts for approximately 80% of lung cancers, and the majority are already unresectable and metastatic upon their initial diagnosis. Cytotoxic chemotherapies such as platinum-based regimens were once the primary therapeutic option for metastatic NSCLC, but their advancement has reached a plateau. Molecular-targeted therapies have been recently developed, and they have provided a remarkable benefit to patients harboring specific genetic alterations such as epidermal growth factor receptor (EGFR) gene mutations or anaplastic lymphoma kinase (ALK) gene fusions [1–3]. Efficacies of up-front EGFR- and ALK-tyrosine kinase inhibitors (TKIs) have been established for patients harboring these genetic alterations in prospective randomized phase III trials comparing platinum doublets, and the median progression-free survivals (PFSs) are approximately 12 months [4–5]. Despite an initial dramatic response, most patients receiving these TKIs finally acquire resistance. Therefore, further salvage therapeutic options are necessary after failure of these molecular-targeted therapies.

On the other hand, current advancement of immunotherapies is evolving. Among them, anti-programmed death-1 (PD-1)/PD-ligand 1 (PD-L1) antibodies have demonstrated their splendid efficacies in pretreated NSCLC. Anti-PD-1/PD-L1 antibodies, such as nivolumab, pembrolizumab, and atezolizumab have shown survival benefit in pretreated patients with NSCLC after failure of platinum doublet chemotherapies, in randomized phase III trials compared to docetaxel monotherapy [6–9]. Based on results of these trials, anti-PD-1/PD-L1 antibody monotherapies have become standard treatments for pretreated NSCLC.

In cases responding to such immunotherapies, durable response is expected over 1-2 years, much longer than common cytotoxic agents [6–9]. Unfortunately, the response rate and PFS of these immunotherapies are generally 10-20% and 2-3 months, respectively, and relatively many patients obtain no response and experience early progression. Notably, several studies demonstrated a possible poorer efficacy of anti-PD-1 antibodies for patients with *EGFR* mutations [7–9]. However, such immunotherapies are not always ineffective even in *EGFR*-mutant NSCLC. Practical predictive markers are necessary to select patients who benefit from anti-PD-1/PD-L1 antibody immunotherapies.

Several predictive markers for anti-PD-1/PD-L1 antibodies have been developed [10]. Among them, PD-L1 expression is the most widely investigated predictive marker for many types of cancers. Some studies for NSCLC have demonstrated correlations between PD-L1 expression and efficacies of anti-PD-1/PD-L1 antibodies [7–9, 11]. One of them has shown that nivolumab was associated with longer overall survival, longer PFS, and higher objective response rates than docetaxel in pretreated NSCLC at the prespecified PD-L1 expression levels of ≥1%, ≥5%, and ≥10% [7].

We thus hypothesized lower PD-L1 expression in *EGFR*-mutant NSCLC samples than in *EGFR*-wild type. The aim of this study was to investigate correlation between PD-L1 expression and *EGFR* mutation status in pretreated NSCLC.

## RESULTS

### Sample and patient profile

**Figure 1:**
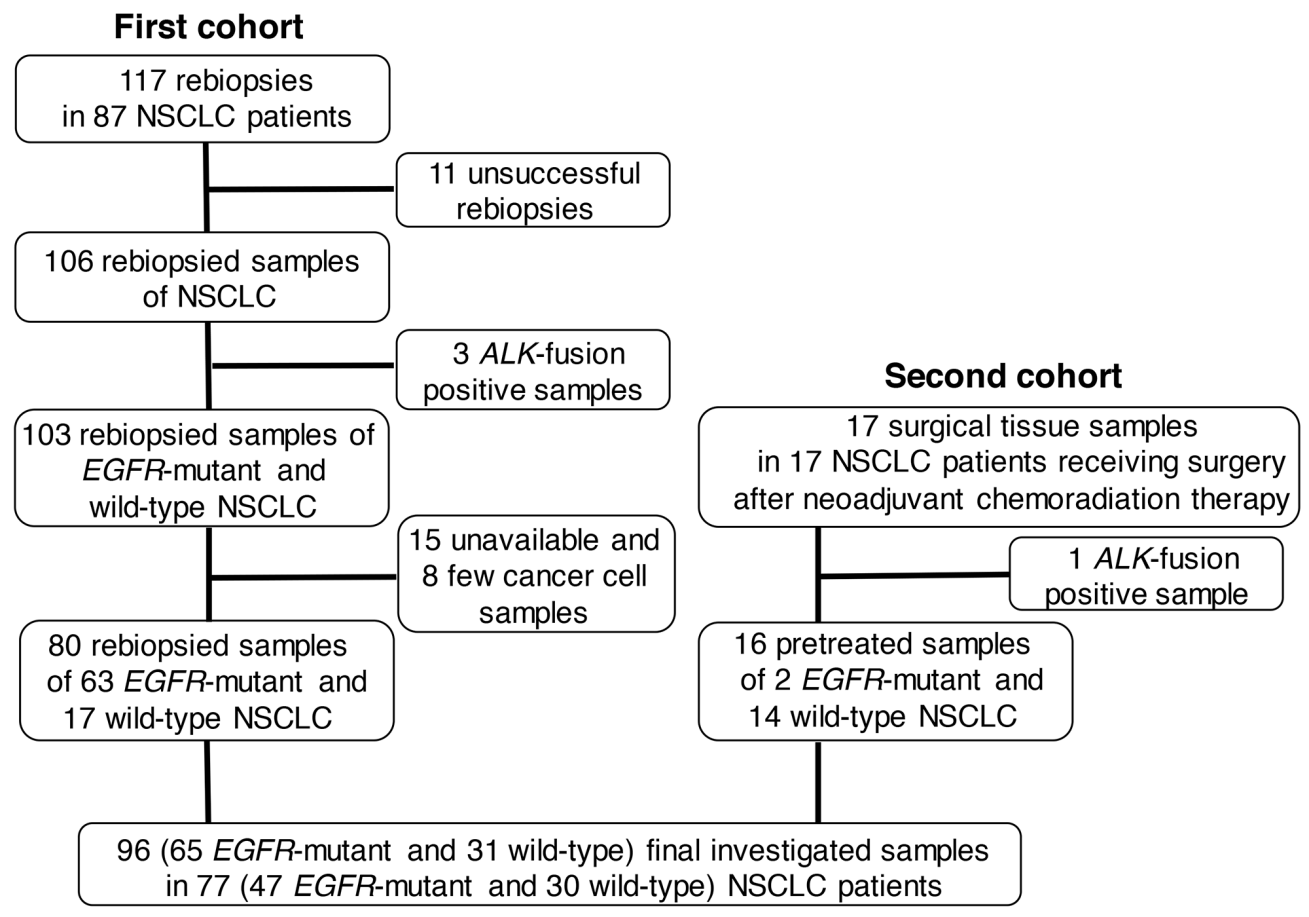
Flow chart of final investigated samples and patients NSCLC, non-small cell lung cancer; ALK, anaplastic lymphoma kinase; EGFR, epidermal growth factor receptor.

Flow chart of final investigated samples and patients is shown in Figure 1. All studied samples were collected between January 2010 and October 2015. In the first cohort, 117 rebiopsies to obtain histologic tissue samples were done in 87 patients with NSCLC. Eleven rebiopsies were unsuccessful and failed to obtain malignant tissue samples. Three *ALK*-fusion positive samples were excluded, and a total 103 *EGFR*-mutant and wild-type tissue samples of NSCLC were examined. Fifteen samples were unavailable, and 8 samples contained insufficient cancer cells to perform PD-L1 IHC. We registered 80 rebiopsied histologic samples of 63 *EGFR*-mutant and wild-type. In the second cohort, 17 surgical tissue samples were available in 17 NSCLC patients receiving surgery after neoadjuvant chemoradiation therapy. After exclusion of one *ALK*-fusion positive sample, 16 of these surgical tissue samples were registered. Finally, we combined pretreated rebiopsy samples and surgical tissue samples, and the number of final investigated samples was 96 (65 *EGFR*-mutant and 31 wild-type) in 77 (47 *EGFR*-mutant and 30 wild-type) NSCLC patients.

Characteristics of final 77 patients investigated are shown in Table 1. Median age was 66 (range, 26-84). Approximately one-third of patients were never smoker. Most tumor histology was adenocarcinoma (63/77, 82%). Types of *EGFR* were: deletional mutation in exon 19 (20/77, 26%); L858R point mutation in exon 21 (25/77, 32%); L861Q point mutation in exon 21 (2/77, 3%), and wild-type (30/77, 39%). Radiotherapy before rebiopsy for sampled tissue was performed in 24 (31%) of patients. Median number of chemo-regimens before rebiopsy was 2 (range, 1–13). Approximately 90% of patients underwent cytotoxic chemotherapies. EGFR-TKIs were prescribed to 47 all *EGFR*-mutant and only 3 *EGFR*-wild-type patients. Rebiopsy was performed to lung lesions in approximately 80% of patients. Extra-lung lesions included: 8 lymph nodes (2 cervical, 2 supraclavicular, 2 axillary, 1 mediastinal, and 1 abdominal); 2 pleural; 2 liver; 2 rib; 1 muscle; and 1 adrenal metastases. Eleven (14%) patients underwent multiple rebiopsies. Median duration from rebiopsy/surgery to PD-L1 IHC evaluation (age of sample) was 21.3 (range, 3.5-71.1) months.

**Table 1:** Patient characteristics (n=77)

Characteristics	Number (%)
Age	
Median (range)	66 (26-84)
<70	48 (62%)
70≤	29 (38%)
Gender	
Male	45 (58%)
Female	32 (42%)
Smoking history	
Never	22 (29%)
Former	27 (35%)
Current	28 (36%)
Histology (initial rebiopsy)	
Adenocarcinoma	63 (82%)
Squamous/Large	11/3 (18%)
Types of *EGFR* mutation	
Exon 19 (deletion)	20 (26%)
Exon 21 (L858R)	25 (32%)
Exon 21 (L861Q)	2 (3%)
Wild-type	30 (39%)
Radiotherapy before rebiopsy for sampled tissue	
Irradiated	25 (32%)
Non-irradiated	52 (68%)
Number of chemo-regimens before rebiopsy	
Median (range)	2 (1-13)
Cytotoxic chemotherapy before rebiopsy	
Received	67 (87%)
None	10 (13%)
EGFR-TKIs before rebiopsy	
Prescribed	50 (65%)
None	27 (35%)
Rebiopsy site	
Lung	61 (79%)
Extra-lung	16 (21%)
Incidence of rebiopsy	
1	66 (86%)
2/3/4/5	6/3/1/1 (14%)
Age of sample (month)	
Median (range)	21.3 (3.5-71.1)
<12 months	53 (69%)
12 months≤	24 (31%)

EGFR-TKI, epidermal growth factor receptor-tyrosine kinase inhibitor.

### Comparison of PD-L1 expression between *EGFR*-mutant and wild-type samples

**Figure 2:**
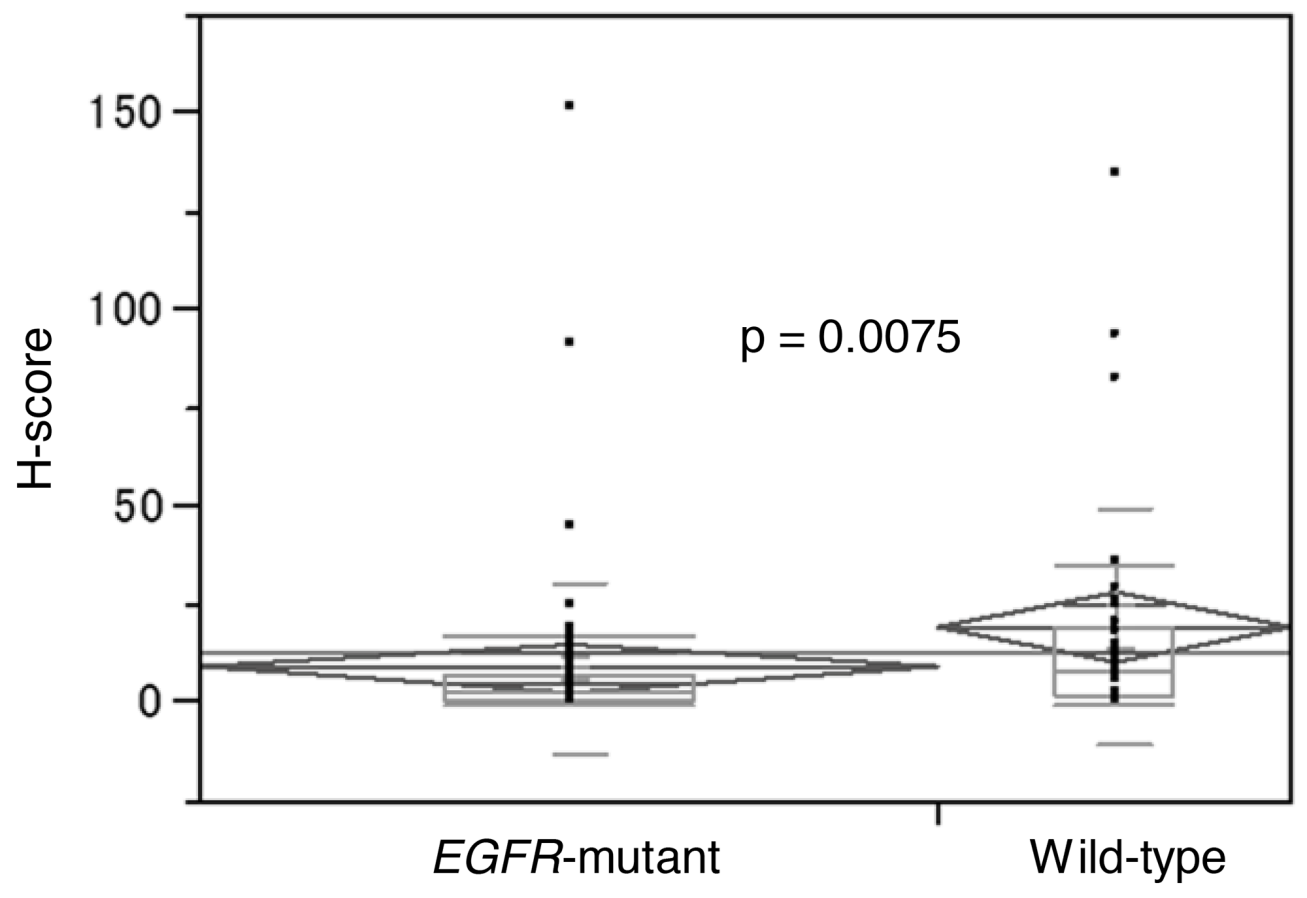
Comparison of H-scores between *EGFR*-mutant and wild-type samples using Wilcoxon rank sum test EGFR, epidermal growth factor receptor.

**Figure 3:**
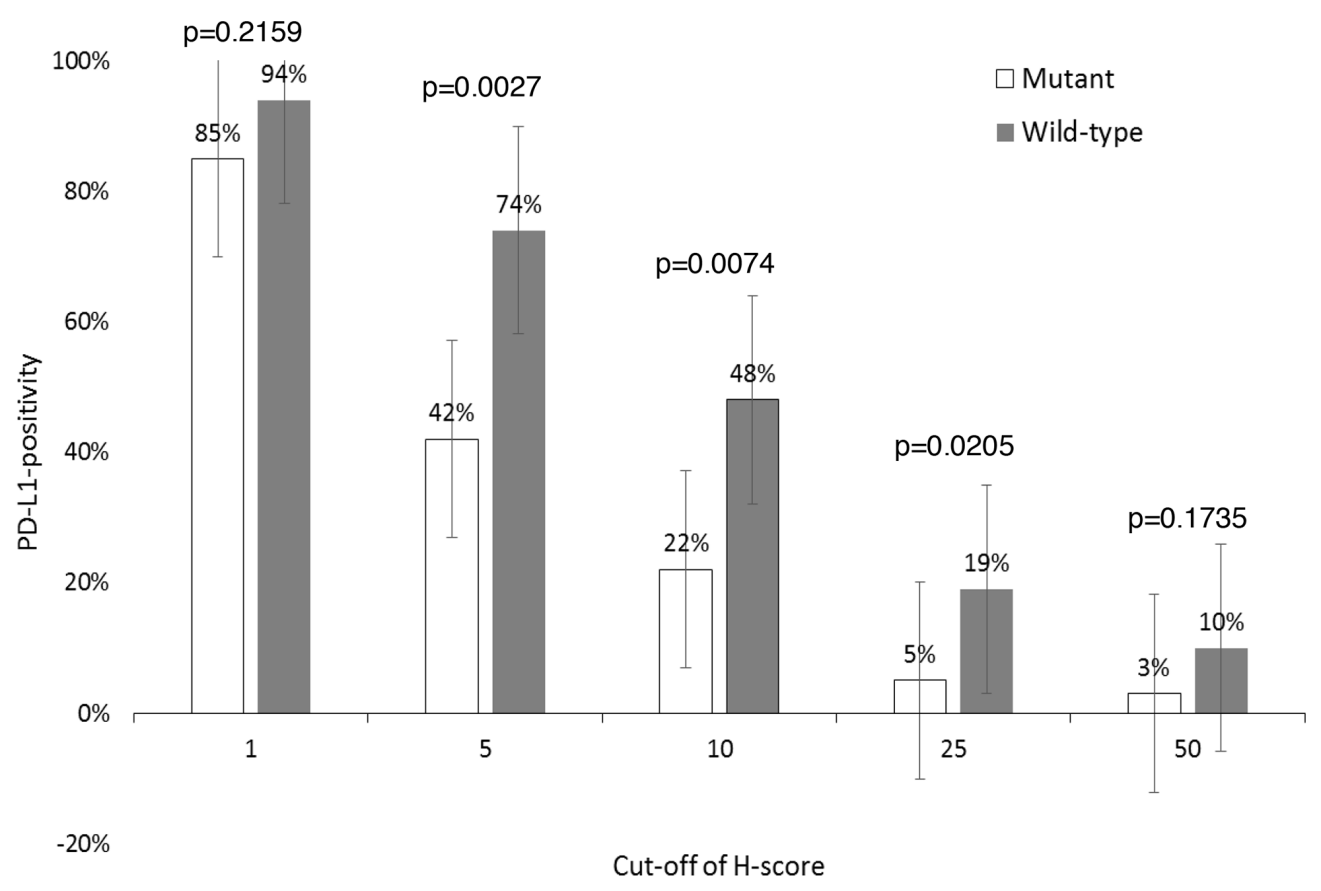
PD-L1-positivity according to *EGFR* status PD-L1, programmed death-ligand 1; EGFR, epidermal growth factor receptor.

**Figure 4:**
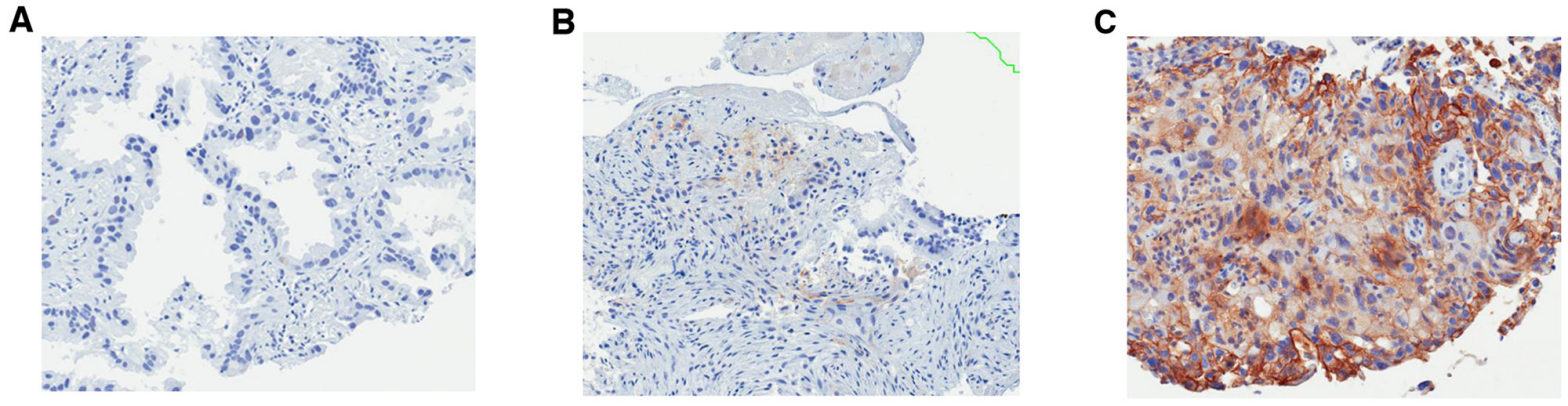
PD-L1 expression of representative samples: PD-L1, programmed death-ligand 1; EGFR, epidermal growth factor receptor **(A)** *EGFR*-mutant (Del-19) (H-score: 0); **(B)** *EGFR*-mutant (L858R) (H-score: 10); **(C)** *EGFR* wild-type (H-score: 134).

Median H-score in *EGFR*-mutant samples (n=65) was 3 (range, 0-150), whereas *EGFR*-wild-type (n=31) was 8 (range, 0-134) (Wilcoxon, p=0.0075) (Figure 2). Using H-scores ≥1, ≥5, ≥10, ≥25, and ≥50 cut-offs, incidence of PD-L1+ in *EGFR*-mutant vs. *EGFR*-wild-type samples were 85% (55/65) vs. 94% (29/31) (p=0.2159), 42% (27/65) vs. 74% (23/31) (p=0.0027), 22% (14/65) vs. 48% (15/31) (p=0.0074), 5% (3/65) vs. 19% (6/31) (p=0.0205), and 3% (2/65) vs. 10% (3/31) (p=0.1735), respectively (Figure 3). Figure 4 shows PD-L1 expression of representative samples: A, *EGFR*-mutant (Del-19) (H-score: 0); B, *EGFR*-mutant (L858R) (H-score: 10); and C, *EGFR* wild-type (H-score: 134).

### Patient-oriented univariate and multivariate analyses for strong PD-L1+

Patient-oriented (n=77) univariate and multivariate analyses for strong PD-L1+ were performed at H-score ≥10 cut-off. In patients receiving multiple rebiopsy, first rebiopsy results were adopted in these analyses. Univariate analysis was performed on: age (<70 vs. 70≤); gender (male vs. female); smoking status (never vs. former vs. current); histology (adeno vs. non-adeno); *EGFR* mutation status (mutant vs. wild-type); radiation before rebiopsy for sampled tissue (irradiated vs. non-irradiated); cytotoxic chemotherapy before rebiopsy (received vs. none) rebiopsy site (lung vs. extra-lung); and age of sample (<12 months vs. 12 months≤). EGFR-TKIs before rebiopsy (prescribed vs. none) was eliminated because of strong confounding to *EGFR* mutation status. Univariate analysis found *EGFR* mutation status (p=0.0490) and age of sample (p=0.0226) as significant factors for strong PD-L1+ (Table 2). Results of multivariate analysis using the logistic regression model are shown in Table 3. We identified *EGFR* status as the only significant factor for strong PD-L1+ (odds ratio, 2.99; 95% confidence interval, 1.34-7.56; and p=0.0121).

**Table 2:** Univariate analysis for strong PD-L1+

Characteristics	PD-L1-positivity (%)	P-value
Age		
<70	20/48 (41.7%)	0.1440
70≤	7/29 (24.1%)	
Gender		
Male	16/45 (35.6%)	NS
Female	11/32 (34.4%)	
Smoking history		
Never	7/22 (31.8%)	NS
Former	10/27 (37.0%)	
Current	10/28 (35.7%)	
Histology (initial rebiopsy)		
Adenocarcinoma	23/63 (36.5%)	0.7592
Squamous/Large	4/14 (28.6%)	
Types of *EGFR* mutation		
Mutant	14/47 (25.5%)	0.0490
Wild-type	15/30 (50.0%)	
Radiotherapy before rebiopsy for sampled tissue		
Irradiated	9/25 (36.0%)	NS
Non-irradiated	18/52 (34.6%)	
Cytotoxic chemotherapy before rebiopsy		
Received	24/67 (35.8%)	NS
None	3/10 (30.0%)	
Rebiopsy site		
Lung	19/61 (31.2%)	0.2384
Extra-lung	8/16 (50.0%)	
Age of sample (month)		
<12 months	14/53 (26.4%)	0.0226
12 months≤	13/24 (54.2%)	

PD-L1, programmed death-ligand 1; EGFR, epidermal growth factor receptor; NS, not significant.

**Table 3:** Multivariate analysis for strong PD-L1+

	Variable	P-value	Odds ratio (95% CI)
Age	(<70 vs. 70≤)	0.1135	0.62 (0.33-1.10)
Smoking history	(Current vs. Former/Never)	0.1063	1.81 (0.92-3.95)
Histology	(Adeno vs. Non-adeno)	0.2268	0.64 (0.27-1.32)
*EGFR* mutation status	(Mutant vs. Wild-type)	0.0121	2.99 (1.34-7.56)
Radiation	(Irradiated vs. Non-irradiated)	0.1867	1.59 (0.83-3.33)
Age of sample	(<12 months vs. 12 months≤)	0.2408	1.41 (0.77-2.51)

PD-L1, programmed death-ligand 1; EGFR, epidermal growth factor receptor; CI, confidence interval.

### PD-L1 expression in multiple rebiopsied cases

Eleven (14%) patients underwent multiple rebiopsies. H-scores of PD-L1 expression varied in all 11 cases receiving multiple rebiopsies (Table 4). PD-L1 expression increased in 7 (64%) patients, whereas decreased in 6 (55%) patients. Categories of positivity: negative (H-score=0); weak+ (1≤ H-score <5); moderate+ (5≤ H-score <10): strong+ (10≤ H-score) migrated in 10 (91%) of 11 patients.

**Table 4:** PD-L1 expression in multiple rebiopsied cases

Case	*EGFR*	Location	H-score at 1^st^ Rebiopsy	H-score at 2^nd^ Rebiopsy	H-score at 3^rd^ Rebiopsy	H-score at 4^th^ Rebiopsy
#1	Mutant	Primary	0	0	0	3
		Metastasis			0	
#2	Mutant	Primary	6			
		Metastasis		2	10	
#3	Mutant	Primary		2	18	
		Metastasis	8			
#4	Mutant	Primary	0	1	2	7
#5	Mutant	Primary	2	1	2	
#6	Mutant	Primary	6	0		
#7	Mutant	Primary	3	5		
#8	Mutant	Primary	1	0		
#9	Mutant	Primary	0	3		
#10	Mutant	Primary	0	1		
#11	Wild	Primary	19	7		

PD-L1, programmed death-ligand 1; EGFR-TKI, epidermal growth factor receptor-tyrosine kinase inhibitor.

## DISCUSSION

We herein demonstrate a lower PD-L1 expression in *EGFR*-mutant NSCLC samples than in *EGFR* wild-type samples. Using H-scores ≥1, ≥5, and ≥10 cut-offs, incidences of PD-L1+ in *EGFR*-mutant samples was also less than in wild-type samples. These results suggest poorer efficacy of anti-PD-1/PD-L1 immunotherapies in *EGFR*-mutant than in wild-type. Gainor et al have reported that NSCLC harboring *EGFR* mutations or *ALK*-fusions were associated with low overall response rate to PD-1/PD-L1 inhibitors [12]. They have also showed lower PD-L1 expression of tissues in these driver oncogene-positive populations than in *EGFR*/*ALK* wild-type population. Our IHC results and their data both support subgroup analyses of clinical studies which showed poorer efficacy of anti-PD-1/PD-L1 immunotherapies in *EGFR*-mutant subgroup [7–9].

Meanwhile, some studies using surgical samples of chemo-naïve NSCLC have shown lower PD-L1 expression in *EGFR*-mutant NSCLC than in *EGFR* wild-type, as we have demonstrated [13, 14]. Others have exhibited higher PD-L1 expression in *EGFR*-mutant NSCLC than in *EGFR* wild-type [15–18]. This issue is still controversial and debatable. Notably, our study and several clinical studies have found a possible temporal heterogeneity of PD-L1 expression by therapeutic interventions, especially EGFR-TKIs [12, 19]. Preclinical studies have also shown that PD-L1 expression was reduced by EGFR-TKIs in NSCLC cell lines harboring *EGFR* activating mutations [20, 21]. Not chemo-naïve surgical samples, but pretreated histological samples (*EGFR*-mutant: after EGFR-TKI therapies) are more desirable for studies to investigate PD-L1 expression. Moreover, anti-PD-1 antibodies, nivolumab and pembrolizumab are approved only in pretreated patients with *EGFR*-mutant NSCLC. To properly investigate this issue, it is preferable to examine pretreated samples for best reflection of study results into clinical practice. Thus, our study focused on histological samples of pretreated NSCLC patients, which is more clinically valuable than studies using chemo-naïve surgical samples.

Our multivariate analysis identified *EGFR* status as the only significant factor for strong PD-L1+. Based on results of pivotal studies regarding nivolumab, PD-L1 expression was associated with clinical efficacies in non-squamous NSCLC population [7]. Although ascertaining PD-L1 expression could be beneficial for non-squamous NSCLC, PD-L1 IHC using 28-8 antibody is uncommon in current clinical practice. We can only utilize clinically available predictive markers. Smoking history and histology might have a predictive value [10, 13, 22], but our multivariate analysis did not reveal a statistical significance. A meta-analysis indicated PD-L1 expression was not associated with common clinicopathological characteristics such as smoking history and histology, except tumor differentiation [23]. Clinical practice demands a routine *EGFR* mutational analysis, which is performed in most cases. Therefore, *EGFR* status could be useful as a predictive marker of anti-PD-1/PD-L1 antibody therapies in pretreated patients with NSCLC.

Age of sample was identified as a significant factor for strong PD-L1+ in our univariate analysis, but multivariate analysis failed to confirm this result. Older samples revealed lesser prevalence of strong PD-L1+. This result implies a possible underestimation of PD-L1 expression in older samples. Several studies suggested that tissue processing and storage could alter the ability to detect PD-L1 in tumor samples [24]. The decreased prevalence may be caused by PD-L1 protein denaturation with formalin fixation and a loss in PD-L1 antigenicity. Age of sample could result in loss of detection of PD-L1 [25]. Based on these studies and our results, rebiopsied fresh samples may be better for PD-L1 IHC.

Issues of PD-L1 IHC contain not only tissue processing and storage but also interpretation of the test by pathologists. Reproducibility is another issue in PD-L1 IHC scoring. We adopted a digital pathological systematic procedure (Aperio). This system can digitally evaluate PD-L1 IHC scores, and demonstrated highly similar IHC staining results to visual evaluation by a pathologist [26]. Our pathologists also confirmed PD-L1 IHC score of each sample, and PD-L1 H-scores were similar between digital procedure and pathologists.

Our study includes several limitations. First is the selection of antibody for PD-L1 IHC. Our study adopted the 28-8 PD-L1 antibody. Four anti PD-L1 antibodies (28-8, 22c3, SP142, and SP263) are clinically used for PD-L1 IHC, but PD-L1 IHC is not globally standardized [27]. Each IHC antibody has been developed simultaneously with each anti-PD-1/PD-L1 therapeutic antibody (nivolumab, pembrolizumab, atezolizumab, and durvalumab). In order to translate basic data for clinical practice, one of these four antibodies for PD-L1 IHC should be used in clinical studies to investigate PD-L1 expression. At present, nivolumab is one of the most widely used immunotherapies in Japan, and thus the 28-8 antibody is an optimal antibody choice for PD-L1 IHC. Second is our cut-offs for PD-L1 positivity. We adopted H-score to evaluate both percentage and intensity, and defined H-scores ≥1 as PD-L1+, scores ≥5 as moderate PD-L1+, and scores ≥10 as strong PD-L1+, largely equivalent to PD-L1 expression ≥1%, ≥5%, and ≥10% cut-offs. Pivotal studies using nivolumab determined PD-L1 expression ≥1%, ≥5%, and ≥10% as their cut-offs [6, 7], and a study for non-squamous NSCLC has demonstrated significant correlation between PD-L1+ status and clinical efficacy of nivolumab [7]. Although optimal cut-off for PD-L1 positivity is yet to be determined, we are sure that our adopted cut-off is appropriate.

In conclusion, our study has demonstrated a significantly lower PD-L1 expression in *EGFR*-mutant NSCLC samples than in *EGFR* wild-type samples. As several studies have shown [7–9], efficacies of anti-PD-1/PD-L1 immunotherapies in *EGFR*-mutant population appear to be poorer than those in *EGFR* wild-type population. Priority of anti-PD-1/PD-L1 immunotherapies might be lower in *EGFR*-mutant population than in *EGFR* wild-type population. Our multiple rebiopsied cases suggested PD-L1 expression dynamism. Age of sample could affect PD-L1 expression, and rebiopsied fresh samples may be better for PD-L1 IHC. Further studies are warranted to investigate association between PD-L1 expression and *EGFR* mutation status.

## MATERIALS AND METHODS

### Samples and patients

We retrospectively screened electronic medical records of patients with NSCLC in our institute. Two sampling cohorts were adopted to collect histological samples in pretreated patients with NSCLC. The first was histological rebiopsied samples after several chemotherapies, regardless of driver oncogene alterations such as *EGFR*/*ALK*. The second was surgical tissue samples after neoadjuvant chemoradiation therapy. After exclusion of samples with *ALK*-fusion, we examined whether each sample contained sufficient cancer cells to perform PD-L1 immunohistochemistry (IHC). After confirmation of cancer cell sufficiency, PD-L1 IHC was carried out. Never smoker was defined as patients who had never smoked in their lifetime. Current smoker was categorized as those who had smoked within 1 year of the diagnosis. The rest were regarded as former smoker. The study was approved by the institutional review board, and complied with the Declaration of Helsinki.

### *EGFR* mutational analysis

We isolated tumor DNA from each specimen, and analyzed *EGFR* mutations using highly sensitive assays: the peptide nucleic acid-locked nucleic acid PCR clamp method [28] or the cycleave method [29].

### PD-L1 immunohistochemistry

Paraffin-embedded tumor tissue was sectioned at a thickness of 4 μm, and the sections were then pasted on coated glass slides for PD-L1 IHC. PD-L1 IHC was performed using the 28-8 antibody for tumor cell membrane staining. Slides were stained with Dako Autostainer Link48. Antigen retrieval was performed in Target Retrieval Solution Low pH. The primary antibody of PD-L1 (clone: 28-8) was diluted at 1:600, and incubated for 45 min at room temperature. The antibody was detected with Rabbit (LINKER) and EnVision FLEX/HRP. Digital image was captured using Aperio Scanscope AT Turbo slide scanner (Leica Biosystems, Vista, CA, USA) under 20x objective magnification. Scoring of PD-L1 was performed using digital image analysis software, namely Aperio membrane v9 and Aperio Genie Classifier. Score of PD-L1 was represented as H-score to evaluate both percentage and intensity. Semiquantitative H-score (maximum value of 300 corresponding to 100% of tumor cells positive for PD-L1 with an overall staining intensity score of 3) was determined by multiplying the percentage of stained cells by an intensity score (0, absent; 1,weak; 2, moderate; and 3, strong). Our pathologists also confirmed PD-L1 IHC score, and no significant difference was found in PD-L1 scores between digital procedure and pathologists. We defined H-scores ≥1 as PD-L1+, scores ≥5 as moderate PD-L1+, and scores ≥10 as strong PD-L1+.

### Statistical analyses

To compare H-scores between *EGFR*-mutant and wild-type samples, we used the Wilcoxon rank sum test. Chi-square test was done to compare the incidence of PD-L1+. Multivariate analysis for strong PD-L1+ was performed using the logistic regression model. Final investigating variables were selected by backward elimination method. A P-value less than 0.05 was considered significant. The statistical analyses were performed using JMP 12 (SAS Institute, Inc., Cary, NC, USA).
